# Surgical Outcomes and Recurrence Management in Borderline Resectable Hepatocellular Carcinoma: Implications for Multidisciplinary Strategies

**DOI:** 10.1002/ags3.70177

**Published:** 2026-01-20

**Authors:** Koichiro Haruki, Kenei Furukawa, Munetoshi Akaoka, Yuto Yamahata, Shinji Onda, Yoshihiro Shirai, Masashi Tsunematsu, Tomohiko Taniai, Michinori Matsumoto, Toru Ikegami

**Affiliations:** ^1^ Division of Hepatobiliary and Pancreatic Surgery, Department of Surgery The Jikei University School of Medicine Minato‐ku Tokyo Japan

**Keywords:** borderline resectable, hepatic resection, hepatocellular carcinoma, prognosis

## Abstract

**Background:**

Oncological resectability classification for hepatocellular carcinoma (HCC) emphasizes the need to reassess treatment strategies for borderline resectable (BR) cases. This study evaluated surgical outcomes and prognostic factors in BR‐HCC and reconsidered multidisciplinary approaches for recurrence management.

**Methods:**

The study comprised 433 patients who underwent primary hepatic resection for HCC between 2000 and 2023. Patients were classified as R, BR1, and BR2 according to the Expert Consensus Statement 2023. Multivariate Cox proportional hazard models were conducted to identify prognostic factors for disease‐free and overall survival in BR (BR1 + BR2) patients.

**Results:**

Among 70 patients with BR‐HCC (BR1: *n* = 37, BR2: *n* = 33), 5‐year overall survival was 53% in BR1 versus 22% in BR2 patients (*p* = 0.06). In multivariate analysis, up‐to‐7 out (*p* < 0.001) and lymph node metastasis (*p* < 0.001) were independent predictors of cancer recurrence, whereas anatomical resection was associated with improved disease‐free survival (*p* = 0.02). Independent predictors of overall survival included older age (*p* = 0.01), Child‐Pugh B (*p* < 0.001), up‐to‐7 out (*p* = 0.01), macrovascular invasion (*p* = 0.01), and lymph node metastasis (*p* < 0.001). Recurrence occurred in 89% of BR‐HCC patients, with BR2 showing more frequent early recurrence within 1 year (*p* = 0.006) and recurrence beyond Milan criteria (*p* = 0.02) compared to BR1. Patients receiving surgical resection or radiofrequency ablation for recurrence demonstrated better survival compared with other treatments (*p* < 0.001).

**Conclusions:**

BR1 and BR2‐HCC represent biologically aggressive subsets with high recurrence rates and poor prognosis, particularly in tumors with up‐to‐7 out status, lymph node metastasis, and macrovascular invasion. Active salvage treatment for recurrence may improve survival, highlighting the importance of multidisciplinary approaches in BR‐HCC management.

## Graphical abstract

Multivariate analysis identified that up‐to‐7 out (*p* < 0.001), lymph node metastasis (*p* < 0.001), and non‐anatomical resection (*p* = 0.02) were independent predictors of cancer recurrence, while older age (*p* = 0.01), Child‐Pugh B (*p* < 0.001), up‐to‐7 out (*p* = 0.01), macrovascular invasion (*p* = 0.01), and lymph node metastasis (*p* < 0.001) were independent predictors of overall survival in patients with borderline resectable (BR1 + BR2) hepatocellular carcinoma after hepatic resection. Moreover, patients receiving repeat resection or radiofrequency ablation for recurrence demonstrated better survival compared with other treatments (*p* < 0.001).

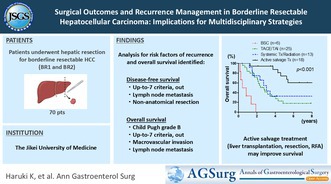

## 1 Introduction

Hepatocellular carcinoma (HCC) is the sixth most common malignancy and third leading cause of cancer‐related mortality worldwide [1]. Among the various therapeutic modalities available, hepatic resection remains the treatment with the highest curative potential for HCC patients. Studies have demonstrated the survival advantages of surgical resection even in advanced HCC cases, including patients with Barcelona Clinic Liver Cancer (BCLC) intermediate stage disease or the presence of vascular invasion and extrahepatic disease in the selected cases [2, 3, 4, 5, 6, 7, 8]. However, the recurrence rate of advanced HCC after curative surgical resection remains very high [9]. Given the advances in systemic therapy, surgical intervention has become a component of the multidisciplinary treatment approach for advanced HCC, and treatment strategies need to be reconsidered [10].

The concept and definition of borderline resectable (BR) HCC has been discussed in terms of biological, technical, and hepatic functional factors [9, 11]. Oncological resectability criteria for HCC has been proposed in the Expert Consensus Statement 2023 in which resectability was defined based on tumor size, tumor number, vascular/bile duct invasion status, and extrahepatic spread [12]. Studies suggested the prognostic utility of this resectability criteria (R, BR1, and BR2) in surgical resection, systemic therapy [13, 14], and status of recurrence [15]. However, due to the limited number of resected cases of BR1 and BR2, prognostic factors in BR (BR1 and BR2) cases have not been fully investigated. Therefore, in this study, we investigated surgical outcomes and prognostic factors in BR (BR1 and BR2)‐HCC and evaluated the multidisciplinary approaches for recurrence management.

## 2 Patients and Methods

### 2.1 Patient Selection

Subjects of this retrospective study were HCC patients who underwent primary hepatic resection at the Department of Surgery, The Jikei University Hospital, Tokyo, Japan between January 2000 and December 2023. We excluded patients who died from other malignancies and postoperative mortality, leaving the remaining 433 patients enrolled in this study. We collected data on clinical information, operative and pathological findings, and postoperative course from medical records. Patients were followed until death or the end of follow‐up. This study was approved by the Ethics Committee of The Jikei University School of Medicine (#27‐177).

### 2.2 Treatment and Follow‐Up

Generally, the extent and type of hepatic resection were determined based on preoperative tumor staging, retention rate of indocyanine green at 15 min (ICG_R15_) before surgery, and hepatic reserve, as previously described [16]. The nomenclature of the segment and types of operations followed the Tokyo 2020 terminology [17]. The type of resection was classified into two groups: anatomical resection (hemihepatectomy, sectionectomy, or segmentectomy) and non‐anatomical limited partial resection [18]. Tumor‐Nodes‐Metastasis (TNM) classification was based on tumor pathology and the General Rules for the Clinical and Pathological Study of Primary Liver Cancer by the Liver Cancer Study Group of Japan [19]. The subject was classified into three groups: R, BR1, and BR2 according to the oncological resectability criteria proposed in the Japan Liver Cancer Association‐Japanese Society of Hepato‐Biliary‐Pancreatic Surgery Expert Consensus Statement 2023 [12].

The recurrence of HCC was defined as newly detected hypervascular hepatic or extrahepatic tumors by ultrasonography, computed tomography, magnetic resonance imaging with or without increase in serum α‐fetoprotein (AFP), or protein induced by vitamin K absence or antagonist‐II.

From January 2000 to February 2018, intrahepatic recurrence was treated by repeated hepatic resection, local ablation therapy, transarterial chemoembolization (TACE), or transarterial infusion (TAI) based on tumor size, number, and location, as well as hepatic functional reserve evaluated mainly by ICG_R15_. Before March 2018, multiple intrahepatic recurrences were mainly treated by TACE or TAI, while since March 2018, lenvatinib and atezolizumab/bevacizumab (since September 2020) have been administered for multiple intrahepatic recurrences instead of TACE and TAI to maintain hepatic functional reserve. Repeated hepatic resection or local ablation therapy was considered if intrahepatic recurrence was basically within the Milan criteria and functionally acceptable for resection or technically accessible for RFA. Extrahepatic recurrence was mainly treated with systemic chemotherapy including sorafenib (since May 2009), lenvatinib, or atezolizumab/bevacizumab, and surgical resection was considered if the extrahepatic lesion was localized. Radiation was given to control localized tumors or bone metastases.

### 2.3 Statistical Analysis

All statistical analyses were conducted using IBM SPSS statistics version 25.0 (IBM Japan, Tokyo, Japan), and all *p*‐values were two‐sided. We used the two‐sided *α* level of 0.05. Our primary analyses were assessment of the prognostic factors for disease‐free and overall survival in borderline resectable HCC. All other tests, including assessment of risk estimates, represented secondary analyses. Data are expressed as a median, interquartile range, or proportion. Continuous and categorical variables were compared using the Mann–Whitney *U*‐test, chi‐squared test, or Fisher's exact test, as appropriate. Overall survival was defined as the time interval from the date of resection to death by any cause, while disease‐free survival was defined as the time interval between the date of resection and the first recurrence or death due to any cause.

We assessed prognostic factors in patients with borderline resectable HCC. Univariable and multivariable Cox proportional hazards regression models were used to estimate hazard ratio (HR) for disease‐free and overall survival. The multivariable Cox regression model initially included age (≥ 65 vs. < 65 years), gender (female vs. male), period (late vs. early), HBsAg status (positive vs. negative), HCVAb status (positive vs. negative), preoperative treatment (yes vs. no), Child‐Pugh grade (B vs. A), ICGR15 (≥ 15 vs. < 15%), serum AFP level (≥ 12 vs. < 12 ng/mL), serum PIVKA‐II level (≥ 315 vs. < 315 mAU/mL), tumor differentiation (poor vs. well/moderate), up‐to‐7 criteria (out vs. in), macrovascular invasion (yes vs. no), lymph node metastasis (yes vs. no), distant metastasis (yes vs. no), oncological resectability (BR2 vs. BR1), and type of resection (anatomical vs. partial). A backward elimination was conducted with a threshold *p* of 0.05 to select variables for the final model. The cut‐off values for continuous variables were defined according to the previous study [9].

The Kaplan–Meier method was used to estimate cumulative survival probabilities, and the differences between groups were compared using the log‐rank test.

## 3 Results

### 3.1 Patient Characteristics

**FIGURE 1:**
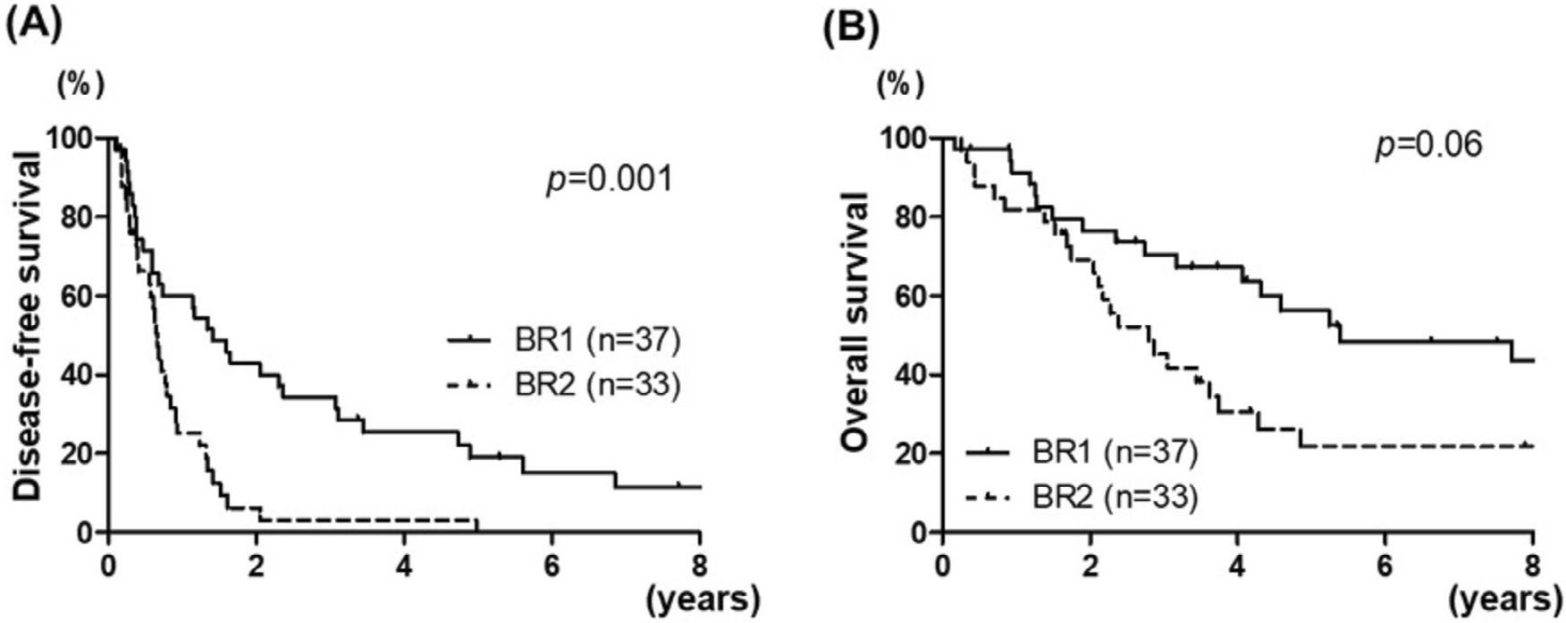
Kaplan–Meier curves of disease‐free (A) and overall survival (B) after hepatic resection for borderline resectable hepatocellular carcinoma. BR2 cases demonstrated significantly worse disease‐free survival compared to BR1 cases (*p* = 0.001), while overall survival showed a trend toward worse outcomes in BR2 cases (*p* = 0.06).

Patient characteristics are outlined in Table 1 as median, interquartile range (IQR), or proportion. Among 433 patients who underwent initial hepatic resection for HCC, 70 patients were classified as borderline resectable HCC, with 37 patients (53%) as BR1 and 33 patients (47%) as BR2 according to the Expert consensus 2023 criteria, which were assessed preoperatively. During follow‐up, 62 of 70 patients experienced tumor recurrence (89%), and the median time to recurrence following hepatic resection was 0.7 years (IQR, 0.4–1.5 years). In the current study, the 5‐year disease‐free survival rates after hepatic resection for borderline resectable HCC were 15% in BR1 and 0% in BR2 (*p* = 0.001, Figure 1A), while the 5‐year overall survival rates were 53% in BR1 and 22% in BR2, respectively (*p* = 0.06, Figure 1B). Preoperative treatment was administered to 20 patients (29%), including TACE/TAE in 16 patients, lenvatinib in 2 patients, and atezolizumab/bevacizumab in 2 patients. TACE was performed for disease control in 15 patients, while TAE was performed for HCC rupture in one patient. Lenvatinib was administered for disease control in one patient with TACE failure and for one patient with intent to conversion (3 months before resection). Atezolizumab/bevacizumab was administered for disease control with intent to conversion (5 months before resection in each case). Although the number of cases that received systemic therapy was limited, systemic therapy was associated with worse disease‐free survival (*p* = 0.03), but was not associated with overall survival (*p* = 0.65) (Figure S1). Combination ablative therapy was performed in seven patients (10%).

**TABLE 1:** Patients characteristics according to the oncological resectability criteria of Expert consensus 2023.

Variables	Total (*n* = 70)	Expert consensus 2023		*p* ^a^
		BR1 (*n* = 37)	BR2 (*n* = 33)	
Age (years)	67 (55–73)	67 (56–73)	70 (53–75)	0.89
Gender				0.68
Female	6 (8.6%)	4 (11%)	2 (6.1%)	
Male	64 (91%)	33 (89%)	31 (94%)	
Period				0.53
Late (May 2009–2023)	52 (74%)	26 (70%)	26 (79%)	
Early (2000–April 2009)	18 (26%)	11 (30%)	7 (21%)	
HBsAg, positive	12 (17%)	5 (14%)	7 (21%)	0.53
HCVAb, positive	15 (22%)	8 (22%)	7 (22%)	0.99
ICG_R15_ (%)	14 (8–20)	12 (8–18)	16 (11–21)	0.10
Child‐Pugh grade				0.99
Grade B	8 (11%)	4 (11%)	4 (12%)	
Grade A	62 (89%)	33 (89%)	29 (88%)	
Preoperative treatment, yes	20 (29%)	8 (22%)	12 (36%)	0.20
Serum AFP (ng/mL)	13 (5–252)	10 (5–246)	44 (6–524)	0.45
Serum PIVKA‐II (mAU/mL)	722 (51–5053)	156 (26–870)	2474 (415–12 844)	0.001
Tumor differentiation				0.51
Poor	11 (16%)	7 (21%)	4 (12%)	
Well/moderate	56 (84%)	27 (79%)	29 (88%)	
Tumor size (cm)	5.6 (4.0–10.5)	4.4 (3.4–7.0)	8.0 (5.7–12)	< 0.001
Tumor number	2 (2–4)	2 (1–4)	4 (2–5)	< 0.001
Up‐to‐7 criteria, out	47 (67%)	15 (41%)	32 (97%)	< 0.001
Macrovascular invasion, yes	17 (24%)	9 (24%)	8 (24%)	0.99
Lymph node metastasis, yes	4 (5.7%)	2 (5.4%)	2 (6.1%)	0.99
Distant metastasis, yes	3 (4.3%)	3 (8.1%)	0 (0%)	0.24
BCLC				
Intermediate	49 (70%)	24 (65%)	25 (76%)	0.43
Advanced	21 (30%)	13 (35%)	8 (24%)	
Type of resection				0.38
Anatomical resection	56 (80%)	28 (76%)	28 (85%)	
Partial resection	14 (20%)	9 (24%)	5 (15%)	
Combination of ablative therapy, yes	7 (10%)	2 (5.4%)	5 (15%)	0.24
Duration of operation (min)	362 (287–529)	354 (282–515)	370 (298–640)	0.33
Intraoperative blood loss (g)	853 (316–1822)	750 (298–1624)	980 (320–2014)	0.46

Abbreviations: AFP, alpha‐fetoprotein; BCLC, Barcelona Clinic Liver Cancer; BR, borderline resectable; HBsAg, hepatitis B surface antigen; HCV‐Ab, hepatitis C virus antibody; ICG_R15_, retention rate of indocyanine green at 15 min; PIVKA‐II, protein induced by vitamin K absence or antagonist‐II.

^a^ To compare categorical data between BR1 and BR2, the chi‐squared test or Fisher's exact test was performed. To compare continuous variables, the Mann–Whitney *U*‐test was performed.

BR2 patients had significantly higher PIVKA‐II levels (*p* = 0.001), larger tumors (*p* < 0.001), greater number of tumors (*p* < 0.001), and greater up‐to‐7 out cases (*p* < 0.001) compared to BR1 patients. Presence of macrovascular invasion, lymph node metastasis, and distant metastasis were comparable between BR1 and BR2 patients (*p* > 0.05).

### 3.2 Univariate and Multivariate Analyses of Clinicopathological Variables Associated With Disease‐Free Survival After Hepatic Resection for Borderline Resectable HCC

**FIGURE 2:**
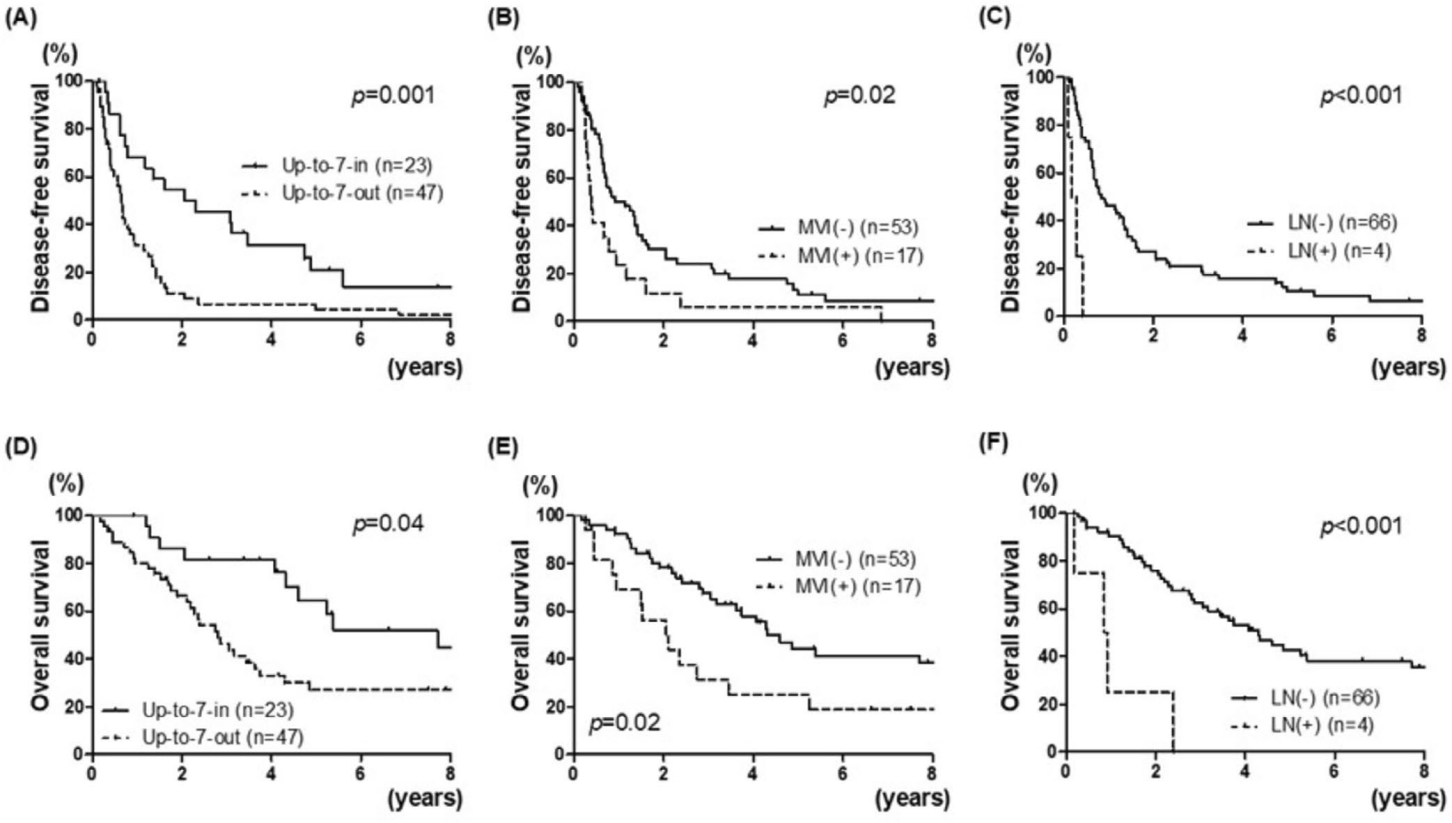
Kaplan–Meier curves of disease‐free (A–C) and overall survival (D–F) after hepatic resection for borderline resectable hepatocellular carcinoma according to up‐to‐7 criteria [(A, D) *p* = 0.001 and *p* = 0.04, respectively], macrovascular invasion [(B, E) *p* = 0.02 and *p* = 0.02, respectively], and lymph node metastasis [(C, F) *p* < 0.001 and *p* < 0.001].

Table 2 lists the association of the clinical variables with disease‐free survival after hepatic resection for borderline resectable HCC. In univariate analysis, disease‐free survival was significantly associated with serum AFP ≥ 12 ng/mL (*p* = 0.003), serum PIVKA‐II level ≥ 315 mAU/mL (*p* = 0.02), up‐to‐7 criteria out (*p* = 0.002, Figure 2A), macrovascular invasion (*p* = 0.047, Figure 2B), lymph node metastasis (*p* < 0.001, Figure 2C), BR2 (*p* = 0.001), and anatomical resection (*p* = 0.01). In multivariate analysis, up‐to‐7 criteria out (*p* < 0.001), lymph node metastasis (*p* < 0.001), and anatomical resection (*p* = 0.02) were independent predictors of disease‐free survival. HRs (95% confidence intervals [CIs]) for up‐to‐7 criteria out, lymph node metastasis, and anatomical resection were 9.05 (2.78–29.44), 4.29 (2.15–8.59), and 0.40 (0.19–0.85), respectively.

**TABLE 2:** Univariate and multivariate analyses of clinicopathological variables associated with disease‐free survival after hepatic resection for borderline resectable hepatocellular carcinoma.

Variables	Univariate analysis		Multivariate analysis	
	HR (95% CI)	*p*	HR (95% CI)	*p* ^a^
Age ≥ 65 years	1.22 (0.72–2.05)	0.47		NS
Gender, female	0.95 (0.38–2.37)	0.90		NS
Period, late	1.30 (0.72–2.34)	0.38		NS
HBsAg, positive	1.05 (0.53–2.08)	0.89		NS
HCVAb, positive	1.23 (0.91–1.67)	0.18		NS
ICG_R15_ ≥ 15%	0.98 (0.59–1.62)	0.94		NS
Child‐Pugh grade, grade B	0.87 (0.35–2.18)	0.76		NS
Preoperative treatment, yes	1.39 (0.80–2.42)	0.24		NS
Tumor differentiation, poor	1.32 (0.65–2.69)	0.44		NS
Serum AFP ≥ 12 ng/mL	2.20 (1.30–3.72)	0.003		NS
Serum PIVKA‐II level ≥ 315 mAU/mL	1.87 (1.08–3.21)	0.02		NS
Up‐to‐7 criteria, out	2.41 (1.37–4.26)	0.002	9.05 (2.78–29.44)	< 0.001
Macrovascular invasion, yes	1.80 (1.01–3.20)	0.047		NS
Lymph node metastasis, yes	8.46 (2.79–25.70)	< 0.001	4.29 (2.15–8.59)	< 0.001
Distant metastasis, yes	1.72 (0.42–7.16)	0.45		NS
Oncological resectability, BR2	2.50 (1.45–4.31)	0.001		NS
Type of resection, anatomical	0.43 (0.23–0.82)	0.01	0.40 (0.19–0.85)	0.02

Abbreviations: AFP, alpha‐fetoprotein; BR, borderline resectable; CI, confidence interval; HBsAg, hepatitis B surface antigen; HCV‐Ab, hepatitis C virus antibody; HR, hazard ratio; ICG_R15_, retention rate of indocyanine green at 15 min; PIVKA‐II, protein induced by vitamin K absence or antagonist‐II.

^a^ The multivariable Cox regression model initially included age (≥ 65 vs. < 65 years), gender (female vs. male), period (late vs. early), HBsAg status (positive vs. negative), HCVAb status (positive vs. negative), preoperative treatment (yes vs. no), Child‐Pugh grade (B vs. A), ICG_R15_ (≥ 15 vs. < 15%), serum AFP level (≥ 12 vs. < 12 ng/mL), serum PIVKA‐II level (≥ 315 vs. < 315 mAU/mL), tumor differentiation (poor vs. well/moderate), up‐to‐7 criteria (out vs. in), macrovascular invasion (yes vs. no), lymph node metastasis (yes vs. no), distant metastasis (yes vs. no), oncological resectability (BR2 vs. BR1), and type of resection (anatomical vs. partial). A backward elimination was conducted with a threshold *p* of 0.05 to select variables for the final model.

Given the advances in systemic therapy, sensitivity analysis was performed using cases after May 2009 (introduction of sorafenib). In the sensitivity analysis, up‐to‐7 criteria out (*p* < 0.001), lymph node metastasis (*p* < 0.001), and anatomical resection (*p* = 0.003) were independent predictors of disease‐free survival (Table S1).

### 3.3 Univariate and Multivariate Analyses of Clinicopathological Variables Associated With Overall Survival After Hepatic Resection for Borderline Resectable HCC

Table 3 lists the association of the clinical variables with overall survival after hepatic resection for borderline resectable HCC. In univariate analysis, the overall survival was significantly associated with Child‐Pugh grade B (*p* = 0.006), serum AFP ≥ 12 ng/mL (*p* = 0.02), up‐to‐7 criteria out (*p* = 0.04, Figure 2D), macrovascular invasion (*p* = 0.02, Figure 2E), and lymph node metastasis (*p* < 0.001, Figure 2F). In multivariate analysis, age ≥ 65 years (*p* = 0.01), Child‐Pugh grade B (*p* = 0.001), up‐to‐7 criteria out (*p* = 0.01), macrovascular invasion (*p* = 0.01), and lymph node metastasis (*p* < 0.001) were independent predictors of overall survival. HRs (95% CIs) for Child‐Pugh grade B, up‐to‐7 criteria out, macrovascular invasion, and lymph node metastasis were 6.63 (2.25–19.53), 3.04 (1.31–7.07), 2.84 (1.26–6.37), and 10.78 (2.96–39.32), respectively.

**TABLE 3:** Univariate and multivariate analyses of clinicopathological variables associated with overall survival after hepatic resection for borderline resectable hepatocellular carcinoma.

Variables	Univariate analysis		Multivariate analysis	
	HR (95% CI)	*p*	HR (95% CI)	*p* ^a^
Age ≥ 65 years	1.22 (0.65–2.27)	0.54	2.77 (1.28–6.02)	0.01
Gender, female	0.91 (0.32–2.55)	0.86		NS
Period, late	0.72 (0.38–1.35)	0.30		NS
HBsAg, positive	1.18 (0.86–3.81)	0.12		NS
HCVAb, positive	1.02 (0.71–1.46)	0.91		NS
ICG_R15_ ≥ 15%	1.58 (0.86–2.91)	0.14		NS
Child‐Pugh grade, grade B	3.61 (1.44–9.02)	0.006	6.63 (2.25–19.53)	0.001
Preoperative treatment, yes	1.24 (0.65–2.36)	0.52		NS
Serum AFP ≥ 12 ng/mL	2.11 (1.12–3.96)	0.02		NS
Serum PIVKA‐II level ≥ 315 mAU/mL	1.35 (0.71–2.57)	0.36		NS
Tumor differentiation, poor	2.01 (0.92–4.42)	0.08		NS
Up‐to‐7 criteria, out	1.99 (1.02–3.91)	0.04	3.04 (1.31–7.07)	0.01
Macrovascular invasion, yes	2.22 (1.14–4.32)	0.02	2.84 (1.26–6.37)	0.01
Lymph node metastasis, yes	6.93 (2.33–20.57)	< 0.001	10.78 (2.96–39.32)	< 0.001
Distant metastasis, yes	0.53 (0.97–3.89)	0.54		NS
Oncological resectability, BR2	1.76 (0.96–3.23)	0.07		NS
Type of resection, anatomical	0.63 (0.32–1.26)	0.19		NS

Abbreviations: AFP, alpha‐fetoprotein; BR, borderline resectable; CI, confidence interval; HBsAg, hepatitis B surface antigen; HCV‐Ab, hepatitis C virus antibody; HR, hazard ratio; ICG_R15_, retention rate of indocyanine green at 15 min; PIVKA‐II, protein induced by vitamin K absence or antagonist‐II.

^a^ The multivariable Cox regression model initially included age (≥ 65 vs. < 65 years), gender (female vs. male), period (late vs. early), HBsAg status (positive vs. negative), HCVAb status (positive vs. negative), preoperative treatment (yes vs. no), Child‐Pugh grade (B vs. A), ICG_R15_ (≥ 15 vs. < 15%), serum AFP level (≥ 12 vs. < 12 ng/mL), serum PIVKA‐II level (≥ 315 vs. < 315 mAU/mL), tumor differentiation (poor vs. well/moderate), up‐to‐7 criteria (out vs. in), macrovascular invasion (yes vs. no), lymph node metastasis (yes vs. no), distant metastasis (yes vs. no), oncological resectability (BR2 vs. BR1), and type of resection (anatomical vs. partial). A backward elimination was conducted with a threshold *p* of 0.05 to select variables for the final model.

In the sensitivity analysis using the subset of cases after May 2009, age ≥ 65 years (*p* = 0.01), Child‐Pugh grade B (*p* = 0.003), up‐to‐7 criteria out (*p* = 0.046), macrovascular invasion (*p* = 0.02), lymph node metastasis (*p* < 0.001), and anatomical resection (*p* = 0.03) were independent predictors of overall survival (Table S2).

### 3.4 Patient Outcomes and Treatment for Recurrence According to the Oncological Resectability Criteria

**FIGURE 3:**
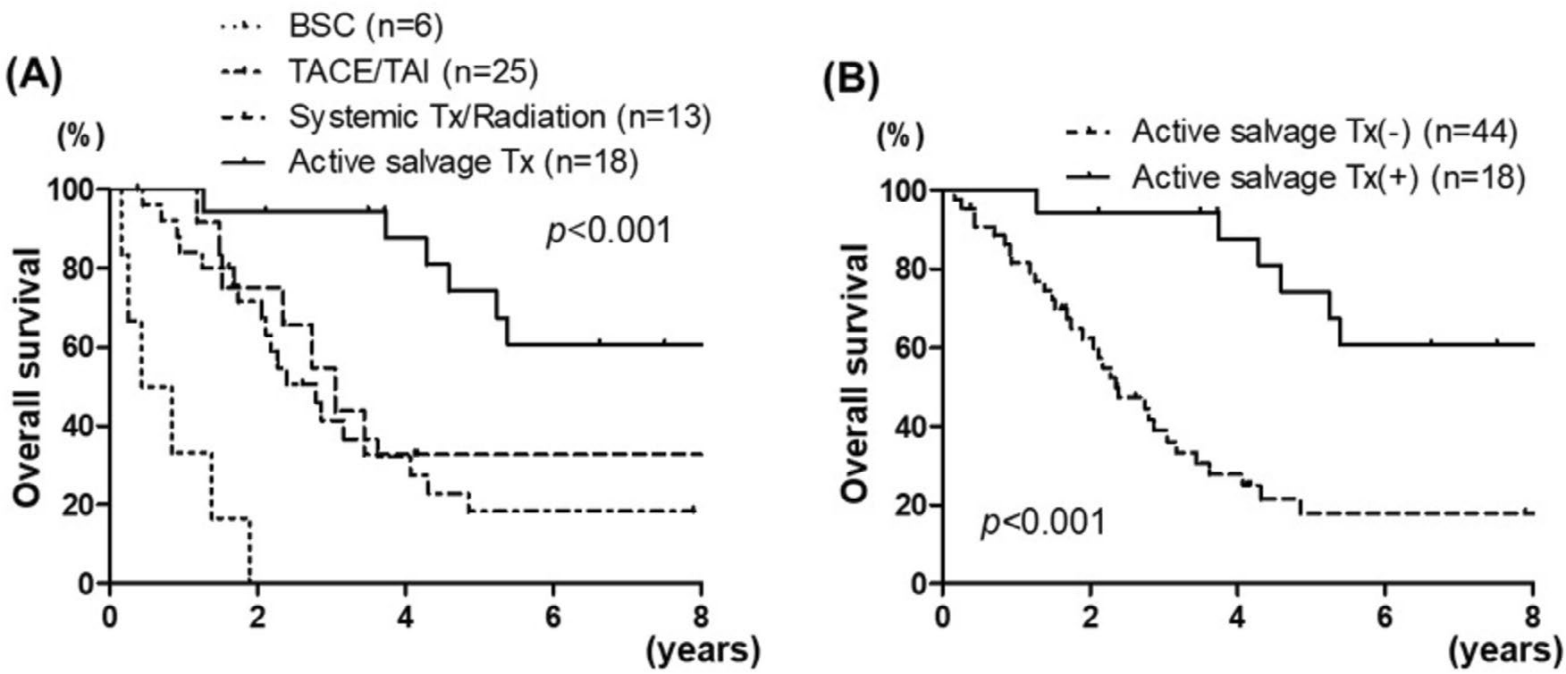
Kaplan–Meier curves of overall survival according to treatment modalities for recurrence after initial hepatic resection for borderline resectable hepatocellular carcinoma. (A) Overall survival by specific treatment types for recurrence (*p* < 0.001). (B) Comparison between active salvage treatments (liver transplantation, surgical resection, and radiofrequency ablation) versus other treatments, demonstrating significantly better survival outcomes with active salvage interventions (*p* < 0.001).

Table 4 lists the patient outcomes and treatment for recurrence according to the Expert consensus 2023 criteria. Early recurrence within 1 year occurred in 38 patients (57%) more frequently in BR2 patients compared to BR1 patients (75% vs. 40%, *p* = 0.006). Recurrence beyond Milan criteria within 1 year was also significantly higher in BR2 patients (56% vs. 29%, *p* = 0.03). Overall recurrence beyond Milan criteria occurred in 38 patients (54%), with BR2 patients showing a higher rate (70% vs. 41%, *p* = 0.02). There were no significant differences in recurrence sites between BR1 and BR2. Treatment options as first‐line therapy included liver transplantation in 1 patient (1.6%), surgical resection in 11 patients (18%), radiofrequency ablation (RFA) in 6 patients (9.7%), systemic therapy in 12 patients (19%), TACE/TAI in 25 patients (40%), radiation in 1 patient (3.3%), and best supportive care in 6 patients (9.7%), with no significant differences between BR1 and BR2 groups (*p* = 0.46). Overall survival differed by treatment for recurrence (*p* < 0.001, Figure 3A) and active salvage treatment including liver transplantation, surgical resection, and RFA demonstrated significantly better survival outcomes compared to other treatments (*p* < 0.001, Figure 3B).

**TABLE 4:** Patients outcomes and treatment for recurrence according to the oncological resectability criteria of Expert consensus 2023.

Variables	Total (*n* = 70)	Expert consensus 2023		*p* ^a^
		BR1 (*n* = 37)	BR2 (*n* = 33)	
Any recurrence, yes	62 (89%)	30 (81%)	32 (97%)	0.06
Recurrence within 1 year				
Any recurrence, yes	38 (57%)	14 (40%)	24 (75%)	0.006
Beyond Milan criteria, yes	28 (42%)	10 (29%)	18 (56%)	0.03
Distant metastasis, yes	14 (21%)	7 (20%)	7 (22%)	0.99
Recurrence beyond Milan criteria	38 (54%)	15 (41%)	23 (70%)	0.02
Recurrence up‐to‐7, out	35 (50%)	15 (41%)	20 (61%)	0.15
Recurrent site				
Liver (including tumor thrombus)	57 (81%)	30 (81%)	27 (82%)	0.99
Lung	8 (11%)	3 (8.1%)	5 (15%)	0.46
Other extrahepatic sites	7 (10%)	3 (8.1%)	4 (12%)	0.70
Treatment for recurrence^b^				0.46
Liver transplantation	1 (1.6%)	1 (1.6%)	0 (0%)	
Resection	11 (18%)	6 (20%)	5 (16%)	
RFA	6 (9.7%)	4 (13%)	2 (6.3%)	
Systemic therapy	12 (19%)	7 (23%)	5 (16%)	
TACE/TAI	25 (40%)	9 (30%)	16 (50%)	
Radiation	1 (3.3%)	0 (0%)	1 (1.6%)	
BSC	6 (9.7%)	2 (6.7%)	4 (13%)	

Abbreviations: BR, borderline resectable; BSC, best supportive care; RFA, radiofrequency ablation; TACE, transarterial chemoembolization; TAI, transarterial injection.

^a^ To compare categorical data between BR1 and BR2, the chi‐squared test or Fisher's exact test was performed. To compare continuous variables, the Mann–Whitney *U*‐test was performed.

^b^ First‐line therapy for recurrence.

### 3.5 Patient Characteristics According to Presence of Active Salvage Treatment for Recurrence

Table 5 lists the patient characteristics according to the presence of active salvage treatment for recurrent disease. Patients who underwent active salvage treatment had significantly fewer primary tumors of up‐to‐7 out (*p* = 0.03) and longer time to recurrence (*p* < 0.001). Patients who did not undergo active salvage treatment were more likely to develop recurrence within 1 year, recurrence beyond Milan criteria within 1 year, distant metastasis, recurrence beyond Milan criteria, or recurrence of up‐to‐7 out tumors. Active salvage treatment was performed for tumors in remnant liver or lung metastasis; however, there was no difference in the distribution of recurrence sites. Early recurrence within 1 year (*p* = 0.001) or recurrence beyond Milan criteria (*p* < 0.001) showed significantly worse overall survival (Figure S2). Surgical resection or RFA was more likely to be performed for late (> 1 year) recurrence and recurrence within Milan criteria (*p* = 0.04 and *p* = 0.001, respectively, Tables S3 and S4).

**TABLE 5:** Patients characteristics according to presence of active salvage treatment for recurrence.

Variables	Active salvage treatment		*p* ^a^
	Yes (*n* = 18)	No (*n* = 44)	
Finding at primary hepatic resection			
Age (years)	66 (56–71)	69 (57–75)	0.10
Gender			0.62
Female	2 (11%)	3 (6.8%)	
Male	16 (89%)	41 (93%)	
Period			0.36
Late (May 2009–2023)	15 (83%)	31 (71%)	
Early (2000–April 2009)	3 (17%)	13 (29%)	
HBsAg, positive	2 (11%)	8 (18%)	0.71
HCVAb, positive	3 (17%)	11 (26%)	0.53
ICG_R15_ (%)	12 (8–16)	15 (10–21)	0.10
Child‐Pugh grade			0.31
Grade B	0 (0%)	5 (11%)	
Grade A	18 (100%)	29 (89%)	
Preoperative treatment, yes	4 (22%)	14 (32%)	0.55
Serum AFP (ng/mL)	10 (6–166)	32 (6–480)	0.59
Serum PIVKA‐II (mAU/mL)	320 (46–1560)	760 (43–6934)	0.28
Tumor differentiation			0.99
Poor	2 (12%)	7 (16%)	
Well/moderate	15 (88%)	36 (84%)	
Tumor size (cm)	5.3 (3.5–8.6)	6.7 (4.4–12)	0.20
Tumor number	3 (2–4)	3 (2–5)	0.78
Up‐to‐7 criteria, out	9 (50%)	35 (80%)	0.03
Macrovascular invasion, yes	3 (17%)	14 (32%)	0.35
Lymph node metastasis, yes	0 (0%)	4 (9.1%)	0.31
Distant metastasis, yes	1 (5.6%)	1 (2.3%)	0.50
BCLC			
Intermediate	15 (83%)	27 (61%)	0.14
Advanced	3 (17%)	17 (39%)	
Oncological resectability			
BR2	7 (39%)	25 (57%)	0.27
BR1	11 (61%)	19 (43%)	
Type of resection			0.31
Anatomical resection	16 (89%)	33 (75%)	
Partial resection	2 (11%)	11 (25%)	
Combination of ablative therapy, yes	2 (11%)	5 (11%)	0.99
Pathological liver cirrhosis, yes	2 (11%)	16 (36%)	0.07
Findings at recurrence			
Time to recurrence	1.6 (0.8–3.2)	0.6 (0.3–1.1)	< 0.001
Recurrence within 1 year			
Any recurrence, yes	5 (28%)	33 (75%)	0.001
Beyond Milan criteria, yes	1 (5.6%)	27 (61%)	< 0.001
Distant metastasis, yes	1 (5.6%)	13 (30%)	0.05
Recurrence beyond Milan criteria	4 (22%)	34 (77%)	< 0.001
Recurrence up‐to‐7, out	2 (11%)	33 (75%)	< 0.001
Recurrent site			
Liver (including tumor thrombus)	16 (89%)	41 (93%)	0.62
Lung	2 (11%)	6 (14%)	0.99
Other extrahepatic sites	0 (0%)	7 (16%)	0.10

Abbreviations: AFP, alpha‐fetoprotein; BCLC, Barcelona Clinic Liver Cancer; BR, borderline resectable; HBsAg, hepatitis B surface antigen; HCV‐Ab, hepatitis C virus antibody; ICG_R15_, retention rate of indocyanine green at 15 min; PIVKA‐II, protein induced by vitamin K absence or antagonist‐II.

^a^ To compare categorical data between groups, the chi‐squared test or Fisher's exact test was performed. To compare continuous variables, the Mann–Whitney *U*‐test was performed.

## 4 Discussion

In the present study, we identified prognostic factors in patients with BR1 and BR2 HCC following surgical resection. Cases exceeding the up‐to‐7 criteria, presence of macrovascular invasion, and lymph node metastasis were associated with unfavorable disease‐free and overall survival, indicating that preoperative systemic therapy may be warranted for such patients. Despite the fact that the majority of BR1 and BR2 cases experienced tumor recurrence, including early recurrence and/or recurrence beyond Milan criteria, active salvage interventions for recurrent disease can improve overall survival. Our findings would help to identify patients who would benefit from surgical resection versus those requiring preoperative systemic therapy in the current era of molecular‐targeted agents.

As oncological resectability category in Expert consensus 2023 has been recently proposed, it would be necessary to evaluate accumulated data on upfront surgery for advanced HCC. Although the oncological resectability categories are defined by tumor size/number, degree of vascular/bile duct invasion, and extent of extrahepatic spread, the prognostic weights of each component in borderline resectable HCC might be different across factors. A study has indicated that the number of criteria fulfilled to define BR2 disease can further stratify oncological outcomes in BR2 HCC patients after surgical resection [13]. In the present study, up‐to‐7‐out tumors, macrovascular invasion, and lymph node metastasis showed unfavorable outcomes after surgical resection. Additionally, oncological resectability categories successfully stratified prognosis and recurrence pattern of patients with BR‐HCC, which was consistent with previous studies [13, 14, 15].

Previous studies have demonstrated the advantages of upfront surgery for advanced HCC with macrovascular invasion and extrahepatic disease in selected patients [2, 3, 4, 5, 6, 7, 8], however, the incidence of early recurrence remains high. Given the therapeutic shift from conventional treatments toward systemic chemotherapy for HCC patients with vascular invasion or multiple tumors in bilobar distribution [20, 21], survival advantages of surgical resection require re‐evaluation as it has become one component of the multidisciplinary treatment approach for advanced HCC [22]. Recent studies indicated the survival benefit of surgical resection for advanced HCC after lenvatinib or atezolizumab plus bevacizumab treatment [23, 24, 25, 26]. Although the number of patients who received preoperative systemic therapy in our cohort was limited, the potential benefits of preoperative systemic therapy warrant further investigation in future treatment strategies. While upfront surgery may be acceptable for a portion of borderline resectable cases, our findings suggest that BR1 patients with adverse prognostic factors such as up‐to‐7‐out tumors, macrovascular invasion, or lymph node metastasis should be considered for preoperative systemic therapy, as these factors were associated with poor prognosis after upfront surgery. Therefore, for BR1 within the up‐to‐7 criteria, upfront surgery can be considered; however, other BR1 tumors and BR2 tumors require systemic therapy, and surgical resection should be considered as a part of the multidisciplinary treatment approach.

Furthermore, the results of this study emphasize the importance of treatment strategies after recurrence. Although the majority of BR1 and BR2 cases experienced early recurrence, patients who underwent active salvage treatments including surgical resection or RFA achieved significantly better survival outcomes. A study has proposed that time‐to‐interventional failure, defined as the time elapsed from resection to unresectable/unablatable recurrence, can be a surrogate marker for overall survival [27]. Another study has demonstrated active salvage treatment has improved survival after recurrence in patients with BCLC‐intermediate and advanced stage HCC [28]. These findings emphasize the critical role of local therapies within the multidisciplinary treatment approach for advanced HCC, suggesting that the focus should not only be on preventing recurrence but also on optimizing treatment strategies for recurrent disease [29]. In our study, treatment strategies for recurrence shifted from TACE/TAI to systemic therapy. In the early period, recurrence was mainly treated with TACE/TAI, which can impair hepatic functional reserve. In contrast, in the late period, active salvage treatment including resection and RFA was performed if intrahepatic recurrence was basically within the Milan criteria with acceptable liver function for resection and technical accessibility for ablative therapy. Although repeated TACE was performed for multiple intrahepatic recurrence in the late period, since 2018, most cases have been treated with systemic therapy. Further studies are needed to evaluate the impact of systemic therapy on recurrent diseases.

Several studies have considered liver function in the assessment of resectability criteria for HCC [11, 23], and it has been an important prognostic factor in HCC. In this study, hepatic functional reserve, represented by Child‐Pugh grade, was not associated with disease‐free survival but was an independent prognostic factor of overall survival. For Child‐Pugh grade B patients, the efficacy of systemic therapy has been limited and TACE may impair liver function. Moreover, studies suggested that impaired hepatic functional reserve has been associated with failure of curative‐intent treatment or shorter time‐to‐treatment failure, resulting in poor prognosis [27, 30]. Although there was not a statistically significant difference, active salvage treatment was more frequently performed in patients with better liver function or non‐cirrhotic liver in the present study.

Several limitations should be acknowledged in this study. First, this was a single‐center retrospective analysis with inherent selection bias. Second, the number of BR cases was relatively limited, which may affect the statistical power of our analyses and precluded detailed analysis of the degree of portal vein invasion, hepatic vein invasion, or lymph node metastasis. Third, the small number of patients who received preoperative systemic therapy limits the evaluation of preoperative treatment. Additionally, oncological resectability was assessed before resection rather than before preoperative treatment. As preoperative exposure time prior to resection was not credited in survival time, this limitation may attenuate observed differences between preoperative treatment and upfront surgery groups. Future studies should evaluate whether preoperative systemic therapy can contribute to down‐staging, reduce recurrence rates, and alter recurrence patterns compared to upfront surgery, particularly focusing on the impact on early recurrence and recurrence beyond Milan criteria that are frequently observed in advanced HCC cases. Forth, intrahepatic metastasis and multicentric occurrence were not distinguished in this study. Active salvage treatment was performed not only for cases with late (i.e., multicentric occurrence) or within Milan criteria recurrence, but also for cases suggesting intrahepatic recurrence (i.e., early or beyond Milan criteria recurrence); however, selection bias in treatment strategy may affect the survival outcomes. Although there has been some evidence that supports our findings, further large‐scale study is necessary to validate the effect of active salvage treatment. Finally, treatment policies and recurrence management strategies evolved considerably over the long study period, especially with the introduction of immune‐based systemic therapies, which may have influenced outcomes. However, given the limited number of patients who received systemic therapy (four patients preoperatively and two patients for recurrence), the impact on our outcomes was likely minimal. We confirmed that sensitivity analyses using subsets of cases in the era of molecular‐targeted therapies did not substantially alter results.

## 5 Conclusions

This study identified key prognostic factors in borderline resectable HCC patients and demonstrated the importance of individualized treatment strategies within a multidisciplinary approach. Up‐to‐7‐out criteria, macrovascular invasion, and lymph node metastasis were independent predictors of poor prognosis, suggesting that even technically resectable patients with these high‐risk features should undergo preoperative systemic therapy with subsequent surgical evaluation, rather than proceeding with upfront surgery. Despite the high recurrence rate in borderline resectable cases, active salvage treatments significantly improved overall survival when functionally and technically feasible, emphasizing the critical importance of optimizing recurrence management strategies.

## Author Contributions

**Koichiro Haruki:** conceptualization, formal analysis, data curation, funding acquisition, writing – original draft, investigation, methodology. **Kenei Furukawa:** data curation, writing – review and editing. **Munetoshi Akaoka:** data curation, writing – review and editing. **Yuto Yamahata:** data curation, writing – review and editing. **Shinji Onda:** data curation, writing – review and editing. **Yoshihiro Shirai:** writing – review and editing. **Masashi Tsunematsu:** writing – review and editing. **Tomohiko Taniai:** data curation, writing – review and editing. **Michinori Matsumoto:** data curation, writing – review and editing. **Toru Ikegami:** writing – review and editing, supervision, funding acquisition.

## Funding

This work was supported by JSPS KAKENHI Grants (24K11920 to K.H. and 24K11898 to T.I.) and research grant from the Takeda Science Foundation (to K.H.). The funders had no role in study design, data collection and analysis, decision to publish, or preparation of the manuscript.

## Ethics Statement

This study protocol was approved by the Ethics Committee of The Jikei University School of Medicine (#27‐177) and it conforms to the provisions of the Declaration of Helsinki.

## Consent

Patients were given an opportunity to opt out of this study through public announcements.

## Conflicts of Interest

The authors declare no conflicts of interest, except that Toru Ikegami serves as an editorial board member of the Annals of Gastroenterological Surgery.

## Supporting information


**Figure S1:** Kaplan–Meier curves of overall survival according to preoperative treatment after hepatic resection for borderline resectable hepatocellular carcinoma.
**Figure S2:** Kaplan–Meier curves of overall survival according to timing and pattern of recurrence after hepatic resection for borderline resectable hepatocellular carcinoma Early recurrence (< 1 year after resection) (A) and beyond Milan criteria recurrence (B) showed worse survival (*p* = 0.001 and *p* < 0.001, respectively).
**Table S1:** Univariate and multivariate analyses of clinicopathological variables associated with disease‐free survival after hepatic resection for borderline resectable hepatocellular carcinoma in the period of 2009–2023.
**Table S2:** Univariate and multivariate analyses of clinicopathological variables associated with overall survival after hepatic resection for borderline resectable hepatocellular carcinoma in the period of 2009–2023.
**Table S3:** Recurrence timing and treatment for recurrence after hepatic resection for borderline resectable hepatocellular carcinoma.
**Table S4:** Recurrence pattern and treatment for recurrence after hepatic resection for borderline resectable hepatocellular carcinoma.
