# Supplementary material for: Surgical Outcomes and Recurrence Management in Borderline Resectable Hepatocellular Carcinoma: Implications for Multidisciplinary Strategies
Source: Ann Gastroenterol Surg. 2026 Jan 20;10(4):1143–54. doi: 10.1002/ags3.70177 (PMC13327078; doi:10.1002/ags3.70177)
Supplement: Supplementary file 1 — Figure S1: Kaplan–Meier curves of overall survival according to preoperative treatment after hepatic resection for borderline resectable hepatocellular carcinoma. Figure S2: Kaplan–Meier curves of overall survival according to timing and pattern of recurrence after hepatic resection for borderline resectable hepatocellular carcinoma Early recurrence (< 1 year after resection) (A) and beyond Milan criteria recurrence (B) showed worse survival (p = 0.001 and p < 0.001, respectively). Table S1: Univariate and multivariate analyses of clinicopathological variables associated with disease‐free survival after hepatic resection for borderline resectable hepatocellular carcinoma in the period of 2009–2023. Table S2: Univariate and multivariate analyses of clinicopathological variables associated with overall survival after hepatic resection for borderline resectable hepatocellular carcinoma in the period of 2009–2023. Table S3: Recurrence timing and treatment for recurrence after hepatic resection for borderline resectable hepatocellular carcinoma. Table S4: Recurrence pattern and treatment for recurrence after hepatic resection for borderline resectable hepatocellular carcinoma. [file AGS3-10-1143-s001.docx]

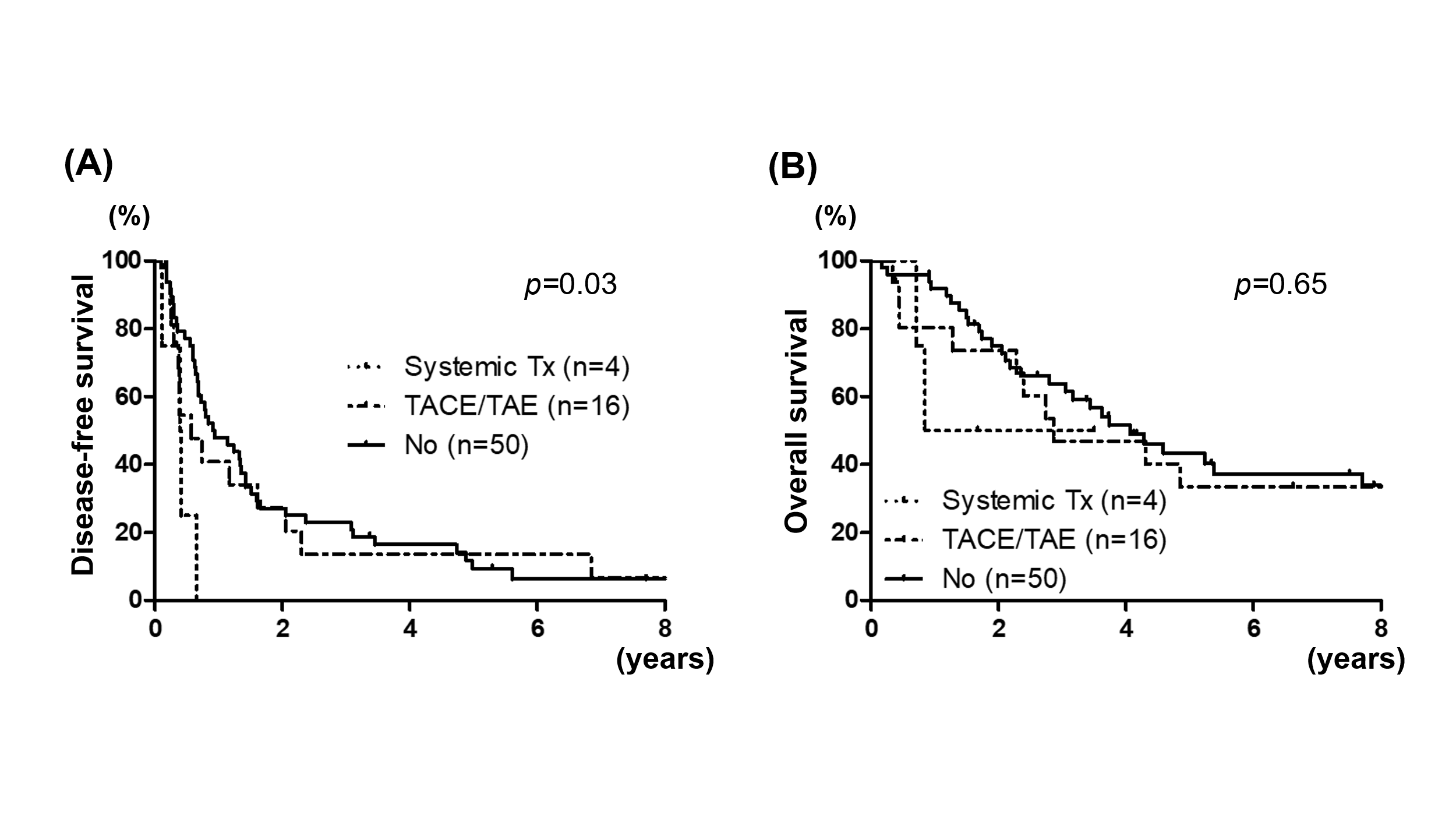


Supplementary Figure 1. Kaplan-Meier curves of overall survival according to preoperative treatment after hepatic resection for borderline resectable hepatocellular carcinoma


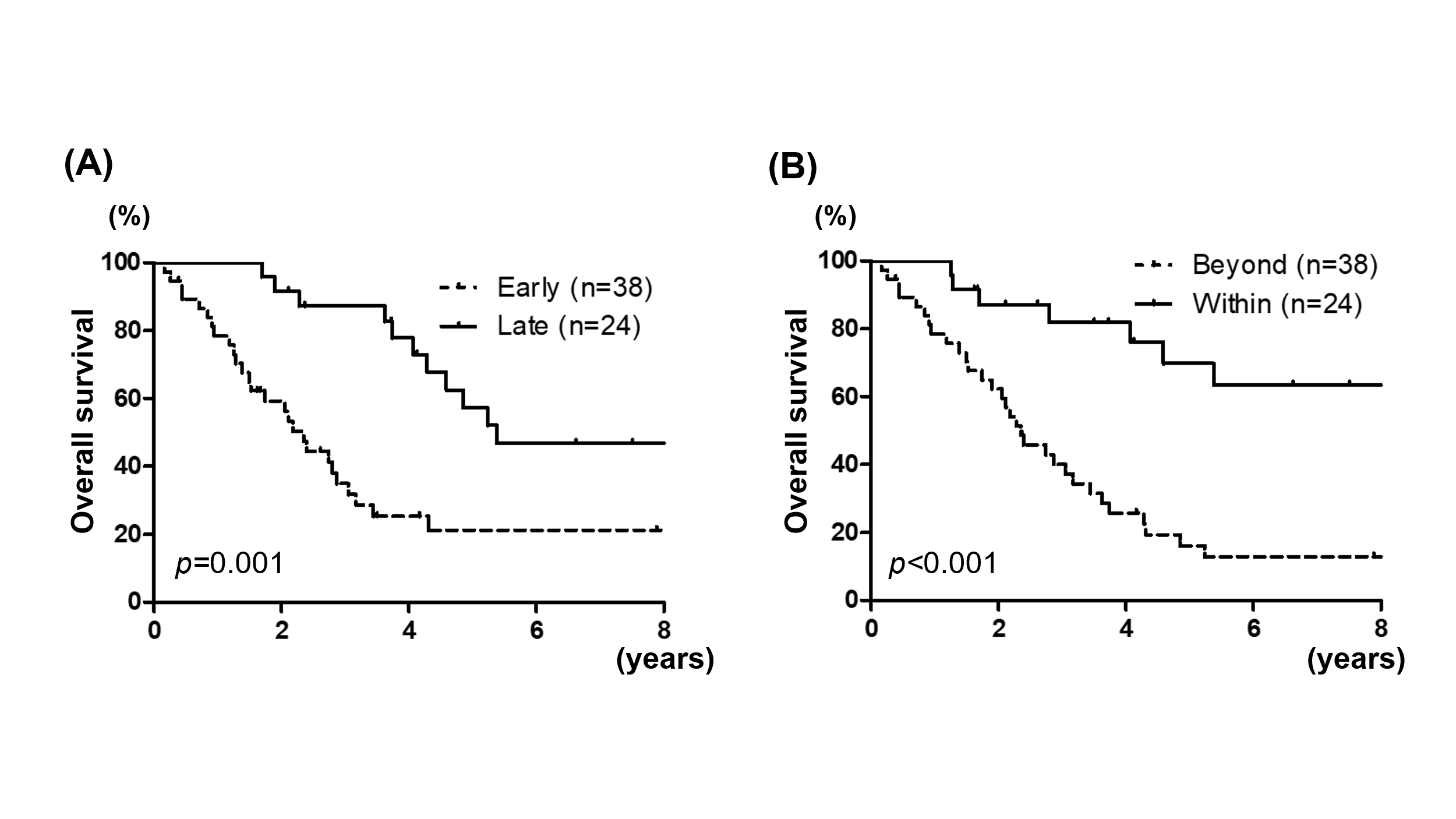


Supplementary Figure 2. Kaplan-Meier curves of overall survival according to timing and pattern of recurrence after hepatic resection for borderline resectable hepatocellular carcinoma Early recurrence (< 1 year after resection) (A) and beyond Milan criteria recurrence (B) showed worse survival (*p*=0.001 and *p*<0.001, respectively).

Supplementary Table 1. Univariate and multivariate analyses of clinicopathological variables associated with disease-free survival after hepatic resection for borderline resectable hepatocellular carcinoma in the period of 2009-2023

| Variables | Univariate analysis | |  | Multivariate analysis | |
| --- | --- | --- | --- | --- | --- |
|  | HR (95% CI) | *p*-value |  | HR (95% CI) | *p*-value* |
| Age ≥ 65 years | 0.98 (0.54-1.78) | 0.94 |  |  | NS |
| Gender, female | 0.77 (0.24-2.51) | 0.67 |  |  | NS |
| HBsAg, positive | 0.70 (0.21-2.33) | 0.56 |  |  | NS |
| HCVAb, positive | 1.24 (0.84-1.83) | 0.28 |  |  | NS |
| ICG_R15_ ≥ 15% | 0.89 (0.50-1.59) | 0.70 |  |  | NS |
| Child-Pugh grade, grade B | 0.95 (0.29-3.10) | 0.94 |  |  | NS |
| Preoperative treatment, yes | 3.03 (1.48-6.19) | 0.002 |  |  | NS |
| Tumor differentiation, poor | 2.51 (1.37-4.61) | 0.003 |  |  | NS |
| Serum AFP ≥ 12 ng/ml | 2.08 (1.10-3.91) | 0.02 |  |  | NS |
| Serum PIVKA-II level ≥ 315 mAU/ml | 1.13 (0.53-2.43) | 0.76 |  |  | NS |
| Up-to-7 criteria, out | 4.30 (2.05-9.02) | <0.001 |  | 3.96 (1.86-8.43) | <0.001 |
| Macrovascular invasion, yes | 2.08 (1.03-4.21) | 0.042 |  |  | NS |
| Lymph node metastasis, yes | 8.11 (2.57-25.61) | <0.001 |  | 9.21 (2.68-31.60) | <0.001 |
| Distant metastasis, yes | 1.04 (0.14-7.65) | 0.97 |  |  | NS |
| Oncological resectability, BR2 | 2.24 (1.19-4.19) | 0.01 |  |  | NS |
| Type of resection, anatomical | 0.29 (0.12-0.72) | 0.007 |  | 0.23 (0.09-0.60) | 0.003 |

Abbreviations: AFP, alpha-fetoprotein; BR, borderline resectable; CI, confidence interval; HBsAg, hepatitis B surface antigen; HCV-Ab, hepatitis C virus antibody; HR, hazard ratio; ICG_R15_, retention rate of indocyanine green at 15 min; PIVKA-II, protein induced by vitamin K absence or antagonist-II.

* The multivariable Cox regression model initially included age (≥ 65 vs. < 65 years), gender (female vs. male), HBsAg status (positive vs. negative), HCVAb status (positive vs. negative), preoperative treatment (yes vs. no), Child-Pugh grade (B vs. A), ICG_R15_ (≥ 15 vs. < 15%), serum AFP level (≥ 12 vs. < 12 ng/ml), serum PIVKA-II level (≥ 315 vs. < 315 mAU/ml), tumor differentiation (poor vs. well/moderate), up-to-7 criteria (out vs. in), macrovascular invasion (yes vs. no), lymph node metastasis (yes vs. no), distant metastasis (yes vs. no), oncological resectability (BR2 vs. BR1), and type of resection (anatomical vs. partial). A backward elimination was conducted with a threshold *p* of 0.05 to select variables for the final model.

Supplementary Table 2. Univariate and multivariate analyses of clinicopathological variables associated with overall survival after hepatic resection for borderline resectable hepatocellular carcinoma in the period of 2009-2023

| Variables | Univariate analysis | |  | Multivariate analysis | |
| --- | --- | --- | --- | --- | --- |
|  | HR (95% CI) | *p*-value |  | HR (95% CI) | *p*-value* |
| Age ≥ 65 years | 1.25 (0.57-2.71) | 0.58 |  | 3.15 (1.29-7.68) | 0.01 |
| Gender, female | 0.67 (0.16-2.84) | 0.59 |  |  | NS |
| HBsAg, positive | 0.69 (0.16-2.92) | 0.61 |  |  | NS |
| HCVAb, positive | 0.89 (0.52-1.51) | 0.66 |  |  | NS |
| ICG_R15_ ≥ 15% | 1.44 (0.68-3.03) | 0.34 |  |  | NS |
| Child-Pugh grade, grade B | 4.08 (1.33-12.53) | 0.01 |  | 7.31 (1.97-27.14) | 0.003 |
| Preoperative treatment, yes | 1.74 (0.76-3.97) | 0.19 |  |  | NS |
| Serum AFP ≥ 12 ng/ml | 2.07 (0.97-4.42) | 0.06 |  |  | NS |
| Serum PIVKA-II level ≥ 315 mAU/ml | 1.31 (0.60-2.84) | 0.50 |  |  | NS |
| Tumor differentiation, poor | 1.89 (0.80-4.49) | 0.15 |  |  | NS |
| Up-to-7 criteria, out | 2.65 (1.07-6.60) | 0.04 |  | 2.76 (1.02-7.48) | 0.046 |
| Macrovascular invasion, yes | 1.94 (0.85-4.45) | 0.12 |  | 2.73 (1.14-6.52) | 0.02 |
| Lymph node metastasis, yes | 8.90 (2.77-28.63) | <0.001 |  | 13.1 (3.19-53.87) | <0.001 |
| Distant metastasis, yes | 0.46 (0.00-322.727) | 0.50 |  |  | NS |
| Oncological resectability, BR2 | 1.45 (0.68-3.08) | 0.33 |  |  | NS |
| Type of resection, anatomical | 0.50 (0.18-1.38) | 0.18 |  | 0.28 (0.09-0.88) | 0.03 |

Abbreviations: AFP, alpha-fetoprotein; BR, borderline resectable; CI, confidence interval; HBsAg, hepatitis B surface antigen; HCV-Ab, hepatitis C virus antibody; HR, hazard ratio; ICG_R15_, retention rate of indocyanine green at 15 min; PIVKA-II, protein induced by vitamin K absence or antagonist-II.

* The multivariable Cox regression model initially included age (≥ 65 vs. < 65 years), gender (female vs. male), HBsAg status (positive vs. negative), HCVAb status (positive vs. negative), preoperative treatment (yes vs. no), Child-Pugh grade (B vs. A), ICG_R15_ (≥ 15 vs. < 15%), serum AFP level (≥ 12 vs. < 12 ng/ml), serum PIVKA-II level (≥ 315 vs. < 315 mAU/ml), tumor differentiation (poor vs. well/moderate), up-to-7 criteria (out vs. in), macrovascular invasion (yes vs. no), lymph node metastasis (yes vs. no), distant metastasis (yes vs. no), oncological resectability (BR2 vs. BR1), and type of resection (anatomical vs. partial). A backward elimination was conducted with a threshold *p* of 0.05 to select variables for the final model.

Supplementary Table 3. Recurrence timing and treatment for recurrence after hepatic resection for borderline resectable hepatocellular carcinoma

| Variables |  | Recurrence | |  |  |
| --- | --- | --- | --- | --- | --- |
|  |  | Early (n=38) | Late (n=24) |  | *p*-value |
| Treatment for recurrence |  |  |  |  | 0.04 |
| Liver transplantation |  | 0 (0%) | 1 (4.2%) |  |  |
| Resection |  | 3 (7.9%) | 8 (33%) |  |  |
| RFA |  | 2 (5.3%) | 4 (17%) |  |  |
| Systemic therapy |  | 9 (24%) | 3 (13%) |  |  |
| TACE/TAI |  | 18 (47%) | 7 (29%) |  |  |
| Radiation |  | 1 (2.6%) | 0 (0%) |  |  |
| BSC |  | 5 (13%) | 1 (4.2%) |  |  |

^†^ To compare categorical data between early and late recurrence, the chi-square test or Fisher's exact test were performed. To compare continuous variables, Mann-Whitney *U*-test was performed.

Abbreviations: BSC, best supportive care; BR, borderline resectable; RFA, radiofrequency ablation; TACE, transcarterial chemo-embolization; TAI, transcarterial injection.

Supplementary Table 4. Recurrence pattern and treatment for recurrence after hepatic resection for borderline resectable hepatocellular carcinoma

| Variables |  | Recurrence | |  |  |
| --- | --- | --- | --- | --- | --- |
|  |  | Beyond Milan criteria (n=38) | Within Milan criteria (n=24) |  | *p*-value |
| Treatment for recurrence |  |  |  |  | 0.001 |
| Liver transplantation |  | 0 (0%) | 1 (4.2%) |  |  |
| Resection |  | 3 (7.9%) | 8 (33%) |  |  |
| RFA |  | 0 (0%) | 6 (25%) |  |  |
| Systemic therapy |  | 10 (26%) | 2 (8.3%) |  |  |
| TACE/TAI |  | 17 (45%) | 8 (33%) |  |  |
| Radiation |  | 1 (2.6%) | 0 (0%) |  |  |
| BSC |  | 6 (16%) | 0 (0%) |  |  |

^†^ To compare categorical data between recurrence beyond and within Milan criteria, the chi-square test or Fisher's exact test were performed. To compare continuous variables, Mann-Whitney *U*-test was performed.

Abbreviations: BSC, best supportive care; BR, borderline resectable; RFA, radiofrequency ablation; TACE, transcarterial chemo-embolization; TAI, transcarterial injection.
